# Horizontal Ridge Augmentation Using a Xenograft Bone Substitute for Implant-Supported Fixed Rehabilitation: A Case Report with Four Years of Follow-Up

**DOI:** 10.1155/2020/6723936

**Published:** 2020-05-14

**Authors:** Bruno Freitas Mello, Márcio de Carvalho Formiga, Luiz Fernando de Souza da Silva, Gustavo dos Santos Coura, Jamil Awad Shibli

**Affiliations:** ^1^Department of Periodontology and Oral Implantology, Dental Research Division, University of Guarulhos, Sao Paulo, Brazil; ^2^Department of Implantology, Unisociesc, Florianópolis, Brazil

## Abstract

The guided bone regeneration (GBR) technique has been used to achieve optimal bone volume augmentation and allow dental implant placement in atrophic maxilla and mandible, with predictable results and high survival rates. The use of bone substitutes has reduced the necessity of autogenous bone grafts, reducing the morbidity at the donor areas and thus improving the patients' satisfaction and comfort. This clinical case report shows a clinical and histological evaluation of the bone tissue behavior, in a case that required the horizontal augmentation of the alveolar ridge, with the use of xenograft biomaterial and further dental implant placement. After six months of healing time, six implants were placed, and a bone biopsy was done. The histological analysis depicted some fragments of the xenograft bone graft, integrated with the new-formed bone tissue.

## 1. Introduction

The rehabilitation of totally, partially, and single-unit edentulous patients with the use of dental implants is an established treatment with high success rates [1, 2]. However, there are several unfavorable clinical situations for the placement of implants, where there is not sufficient bone height and/or width in the alveolar bone ridge. Augmentation of the alveolar ridge using guided bone regeneration (GBR) became a treatment option to obtain bone support for osseointegrated dental implants [3]. The procedures for bone augmentation of the alveolar ridge through GBR present successful, long-term follow-up results [4–6].

Autogenous bone grafts are often used in GBR procedures and are considered as the gold standard because it is the only biomaterial that combines properties of osteogenesis, osteoinduction, and osteoconduction [7, 8]. However, disadvantages such as morbidity at the donor site, limited availability, tooth sensitivity, and risk of dehiscence of the wounds [1, 7, 9–11] have led to investigations on the development and application of bone substitutes for the regeneration of the alveolar bone ridge [11–13].

Xenografts have shown to have excellent properties for GBR, like its biocompatibility, formation of a scaffold (osteoconduction), slow resorption rates, and the ability to define and maintain the volume for bone gain [12–14]. The graft maturation period could be longer than that of autogenous bone grafts, taking from nine to twelve months [10, 12–14], and demand the need of collagen membranes for guided bone regeneration procedures, which must provide cell occlusion and a better biocompatibility with the soft tissue, reducing the risk of complications such as wound dehiscence [11, 14].

Therefore, the aim of this case report is to describe a GBR with xenograft and collagen membrane for horizontal augmentation for an implant-supported fixed rehabilitation and the four years of follow-up.

## 2. Case Report

**Figure 1:**
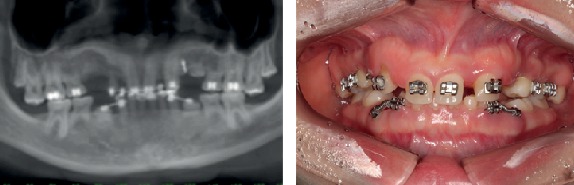
Panoramic radiograph (a) and intraoral buccal view, showing permanent tooth absences and presence of several deciduous teeth.

An 18-year-old female patient attended a clinic/school seeking to solve aesthetic and functional problems. After an initial evaluation, several dental losses and the presence of some deciduous teeth were observed, revealing a multiple agenesis condition. Clinical examination and CT scans were performed. In the mandible, the patient presented all the deciduous teeth, and only permanent teeth present were #36 and #46 (Figure 1).

**Figure 2:**
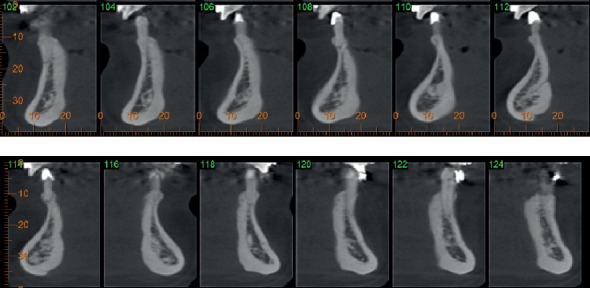
Preoperatory tomography of the anterior mandible region.

Analyzing the mandible CT scan, it was observed a sizeable horizontal deficiency of the alveolar ridge in the crestal symphysis region. This situation made impossible the placement of implants in the ideal three-dimensional position, requiring previous bone reconstruction (Figure 2).

**Figure 3:**
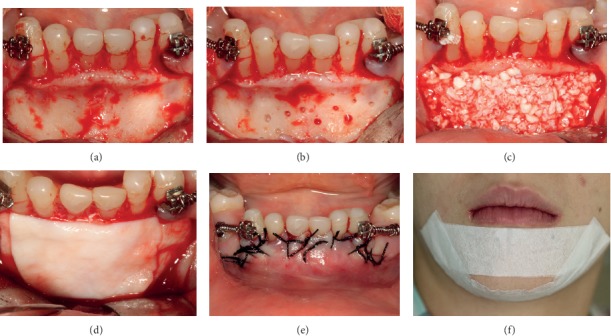
Intraoral view of the surgery steps: (a) incision and total flap detachment, (b) cortical perforations, (c) bone substitute accommodation, (d) double layer collagen membrane, (e) single sutures, and (f) bandage (Micropore®).

It was decided to perform horizontal bone augmentation by guided bone regeneration with only a bovine bone substitute (Bio-Oss® Geistlich), because it presents reproducible results with acceptable success rates and low rate of complications, besides providing lower morbidity for the patient. The marginal gingiva was healthy and presented a good width of keratinized tissue. Amoxicillin 500 mg 1 every 8 hours for 7 days and 0.12% chlorhexidine gluconate rinse 3 times a day for 14 days were prescribed, initiating 2 days before surgery. Infiltration anesthesia was performed with 4% articaine anesthetic in the mental nerve plexus, bilaterally. The incision was intrasulcular in the region from 75 to 85, with small vertical releases at the distal of this incision. A mucoperiosteal flap was raised. At the recipient site, soft tissue debridement and small perforations with 1.3 drills were performed in the cortical bone with the purpose of providing irrigation and blood supply to the bone substitute. At this moment, 1.0 cc of bone substitute (Bio-Oss® Geistlich, large granules) was prepared to be placed at the recipient site and covered with double layer collagen resorbable membrane (Bio-Gide® Geistlich, 25 mm × 25 mm). Both bone substitute and collagen membrane were displaced on different recipients, with saline for 10 minutes of hydration prior use. The buccal flap was repositioned, covering all the membrane and avoiding displacement. The suture was performed with simple interrupted and sling sutures using the deciduous teeth as support, with silk 4.0 (Ethicon). After the surgical procedure, a bandage (Micropore®) was performed to compress the surgical area to diminish possible edema (Figure 3).

Nimesulide 100 mg twice a day for 3 days, paracetamol 750 mg 3 times a day (if necessary for pain control), and continuation of the preoperative antibiotic and antiseptic mouthwash protocols were prescribed. The patient was instructed to eat only soft foods, put ice bags on the surgical area for 48 hours, and avoid exercises for 7 days. The sutures were removed after 7 days.

**Figure 4:**
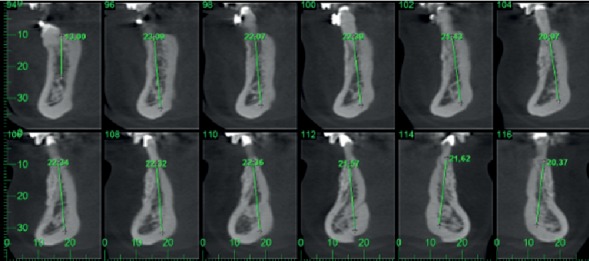
Postoperatory tomography radiograph 12 months after the horizontal bone augmentation, showing new bone formation, allowing the correct three-dimensional placement of dental implants.

**Figure 5:**
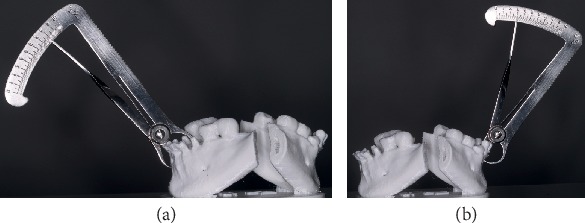
3D resin prototype of the appearance of the ridge before (a) and after (b) the volume increase of the alveolar ridge. There was a 5 mm gain in bone thickness.

After 12 months, the area of the horizontal bone augmentation was clinically evaluated and through cone-beam CT. After the analysis of the tomography radiograph and initial and final prototypes, the gain in bone thickness was approximately 5 mm. Clinically, the success of the procedure was confirmed, as well as the presence of good health of the gingival tissue (Figures 4 and 5).

**Figure 6:**
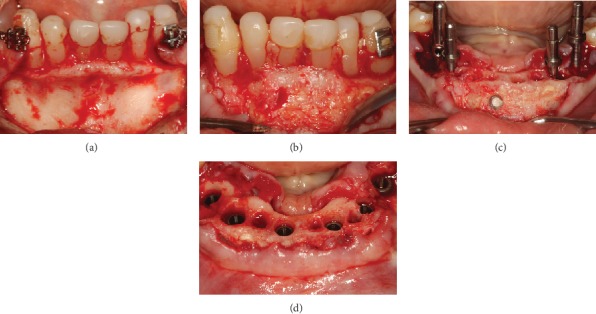
Intraoral view of the alveolar ridge before (a) and after (b) augmentation. Removal of bone fragment using a 2.0 mm trephine for histological analysis of the grafted area (c). Six implants in place with initial torque of 35 N/cm (d).

The surgery to install 6 dental implants (Ankylos®, Dentsply) was performed 12 months following the horizontal bone augmentation, according to the reverse planning and surgical guide, between the region of #34 and #44. All implants presented initial stability above 35 N/cm. The deciduous teeth 74, 73, 72, 71, 81, 82, 83, and 84 were removed at this time. After detachment of the mucoperiosteal flap, we found new bone tissue with excellent quality with the native bone appearance and without the presence of loose particles of a bone substitute and surrounded by fibrous tissue. At this moment, a bone fragment of approximately 3 mm was removed using a 2.0 mm trephine drill in a buccolingual direction for histological analysis of the grafted area (Figure 6).

**Figure 7:**
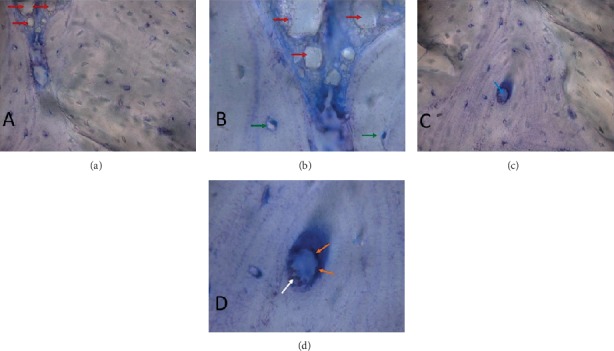
(a) Image of a grafted material interface (red arrow) and cortical bone. It is clearly shown the formation of cortical bone with osteocytes; (b) enlarged image of (a). Grafted material (red arrow). Osteocytes present in the gaps (green arrow); (c) well-formed Haversian system: note the arrangement of concentric lamellae, with the central channel of Havers (blue arrow). Osteocytes homogeneously distributed, demonstrating the usual architecture of cortical bone; (c) enlarged image of (d). In detail, the Havers channel, with endosteum (orange arrow) and red blood cells (white arrow).

In the histological analysis, it was observed the formation of cortical bone with the presence of osteocytes found in several sizes and homogeneously distributed. Bone substitute particles were present in a graft/cortical bone interface. However, it could be confirmed on the clinical observation that the regenerated bone was similar to the natural bone (Figure 7).

**Figure 8:**
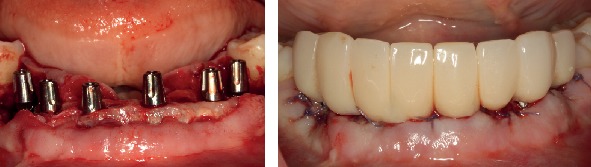
Installation of the definitive prosthetic abutments and delivery of the temporary cemented restoration with immediate load.

Immediately after implant surgery, standard prosthetic abutments (A4) were installed (Ankylos®). After suturing, with 5.0 polypropylene (Atramat), because of its fewer biofilm accumulation, the impression-taking of the abutments and occlusal registration were accomplished. A temporary acrylic resin fixed prosthesis was installed immediately to the surgical procedure, within 24 hours (Figure 8). Pre- and postoperative recommendations and medication protocols were identic to the first surgery, but without the need for bandages.

**Figure 9:**
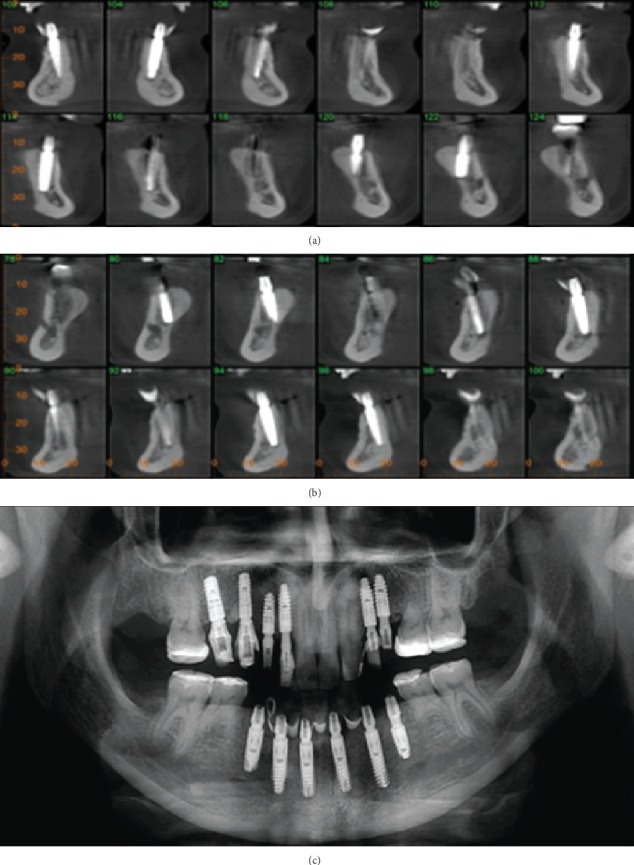
Four-year follow-up CT, showing the stability of the bone tissue around the implants (a, b). Panoramic radiograph of the 4-year follow-up (c).

**Figure 10:**
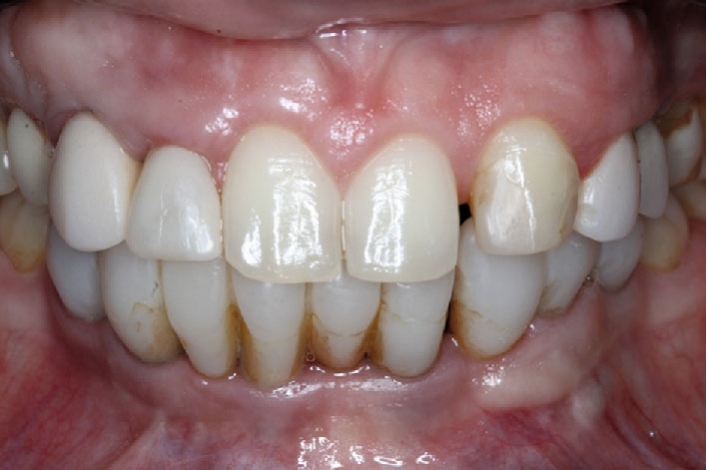
Buccal view of the four-year follow-up.

Four years after bone augmentation of the alveolar ridge, a tomographic examination was requested to evaluate the bone tissue, as well as the implants, where bone normality and stability around the implants were seen, to confirm our clinical evaluation (Figures 9 and 10).

## 3. Discussion

Guided bone regeneration (GBR) performed within the concepts of the technique can be indicated to increase the width of alveolar bone ridges when associated with the use of collagen membranes and bovine bone substitute, with high survival rates of implants placed in sites with large bone volume deficiencies [6, 10, 15].

The horizontal bone gain of about 5 mm after 12 months observed in this clinical case is within the mean gain obtained by other clinical reports of GBR approach in the literature [11, 12]. This bone augmentation provided enough bone width for dental implant placement in an ideal three-dimensional position.

Soft tissue healing was excellent, with no intercurrences, such as membrane exposure or infections. This corroborated further studies on the compatibility of collagen membranes with soft tissue [9–12]. Studies reported that second intention healing occurred in all membrane exposures, with spontaneous reepithelialization, with no major complications [9].

The collagen absorbable membranes, due to its hydrophilic property and excellent interaction with blood clot fibrin in the surgical wound, facilitate the suture procedure [16] and have less tendency to soft tissue dehiscence [14]. In this case, we used silk suture on the GBR procedure because it was more comfortable for the patient, and the patient had excellent biofilm control. We realize it was not the best suture biomaterial, but the patient was very cooperative during all treatment. On the implant surgery, we preferred the use of polypropylene sutures because it remained above the temporary fixed prosthesis, and this material would provide a better biofilm control. To achieve success in the procedures, GBR membranes must possess functional durability, be resistant to degradation, and have biocompatibility with the soft tissue, providing tissue integration and avoiding spaces that may predispose to tissue dehiscence and infections [15]. The disadvantages of the collagen membrane are the resorption period between 4 and 8 weeks and lack of rigidity, leading to the need for filling material under the membrane thus providing the contour for bone augmentation [16].

Nonabsorbable membranes have greater stability in GBR and are more predictable regarding the time of permanence and maintenance of the scaffold in the alveolar ridge, with excellent results for bone augmentation [11, 16]. However, some reports have shown that nonresorbable membranes are more critical about their use, leading to high rates of wound dehiscence with subsequent local exposures and infections, and require a second surgery for its often removal [6, 9, 14].

Xenogenous bovine bone substitutes are composed basically of hydroxyapatite, after a process of elimination of all the organic components, resulting in a structure similar to the human bone. Thus, at the end of this process, a biomaterial should be obtained where its particles may have interconnecting pores allowing thereby the penetration of new blood vessels and the osteoblast cellular migration into the graft [13].

In the literature, there are studies that used GBR protocols using collagen membranes and a mixture of autogenous bone/bone substitute [11]. The authors advocate the use of this mixture by the fact that autogenous bone particles add an osteogenic property to the graft.

However, one of the main advantages of the GBR approach using only bone substitutes is to avoid the complications of autogenous bone removal such as donor area morbidity, limited availability, and tooth sensitivity [1, 7, 9, 10]. Thus, the use of bone substitutes provides more satisfaction and comfort for the patient. The average results of gains in these studies that used autogenous bone/bone substitute mixture [10, 11] are comparable to this clinical case report. Other clinical studies [17, 18] showed similar results, as we found in this case report, with high rates of success on implant survival. Recent reviews also endorse the findings of this case report, with similar rates of horizontal bone augmentation [19–21].

## 4. Conclusion

Within the limitations of this report, the horizontal bone augmentation with the use of only a bovine bone substitute and a double layer collagen membrane seemed to be a feasible treatment option in the short term for promoting an increase in bone width of the alveolar ridge, allowing an appropriate size for predictable and successful placement of dental implants and a fixed oral implant-supported rehabilitation.

## Data Availability

The references used to support the findings of this case report are listed in References and can be found at PubMed.
